# The Application of Artificial Intelligence (AI)-Based Ultrasound for the Diagnosis of Fatty Liver Disease: A Systematic Review

**DOI:** 10.7759/cureus.50601

**Published:** 2023-12-15

**Authors:** Basil N Nduma, Yazan A Al-Ajlouni, Basile Njei

**Affiliations:** 1 Internal Medicine, Merit Health Wesley, Hattiesburg, USA; 2 Medicine, New York Medical College, New York, USA; 3 Medicine, Yale School of Medicine, New Haven, USA

**Keywords:** systematic review, imaging techniques, fatty liver disease diagnosis, artificial intelligence, hepatic steatosis

## Abstract

Fatty liver disease, also known as hepatic steatosis, poses a significant global health concern due to the excessive accumulation of fat within the liver. If left untreated, this condition can give rise to severe complications. Recent advances in artificial intelligence (AI, specifically AI-based ultrasound imaging) offer promising tools for diagnosing this condition. This review endeavors to explore the current state of research concerning AI's role in diagnosing fatty liver disease, with a particular emphasis on imaging methods. To this end, a comprehensive search was conducted across electronic databases, including Google Scholar and Embase, to identify relevant studies published between January 2010 and May 2023, with keywords such as "fatty liver disease" and "artificial intelligence (AI)." The article selection process adhered to the PRISMA framework, ultimately resulting in the inclusion of 13 studies. These studies leveraged AI-assisted ultrasound due to its accessibility and cost-effectiveness, and they hailed from diverse countries, including India, China, Singapore, the United States, Egypt, Iran, Poland, Malaysia, and Korea. These studies employed a variety of AI classifiers, such as support vector machines, convolutional neural networks, multilayer perceptron, fuzzy Sugeno, and probabilistic neural networks, all of which demonstrated a remarkable level of precision. Notably, one study even achieved a diagnostic accuracy rate of 100%, underscoring AI's potential in diagnosing fatty liver disease. Nevertheless, the review acknowledged certain limitations within the included studies, with the majority featuring relatively small sample sizes, often encompassing fewer than 100 patients. Additionally, the variability in AI algorithms and imaging techniques added complexity to the comparative analysis. In conclusion, this review emphasizes the potential of AI in enhancing the diagnosis and management of fatty liver disease through advanced imaging techniques. Future research endeavors should prioritize the execution of large-scale studies that employ standardized AI algorithms and imaging techniques to validate AI's utility in diagnosing this prevalent health condition.

## Introduction and background

Fatty liver disease, characterized by fat accumulation in the liver, has two primary variants: alcoholic fatty liver disease, or steatohepatitis, and non-alcoholic fatty liver disease (NAFLD). The latter, which is unrelated to excessive alcohol consumption, can be divided into two categories. First, there's a simple fatty liver, characterized by fat accumulation without significant hepatocyte damage or inflammation. The second category is non-alcoholic steatohepatitis (NASH), which involves fat buildup, hepatocellular injury, and inflammation [1].

Conversely, alcoholic steatohepatitis is a consequence of heavy alcohol consumption. It results from an immune system response to toxic metabolites generated during hepatic alcohol metabolism, leading to inflammatory harm to hepatocytes [2]. This spectrum ranges from alcoholic fatty liver disease to alcoholic hepatitis and cirrhosis [1,2].

The global landscape bears witness to an alarming escalation in fatty liver disease prevalence. It is important to note that the prevalence varies widely depending on the population studied and the definition used. However, it is estimated that NAFLD affects a quarter of the world's population [3]. Additionally, nearly half of the afflicted individuals in the United States are middle-aged [3]. Furthermore, estimates indicate that 20% to 30% of newly diagnosed NAFLD cases may have progressed to NASH, with a subsequent 10% to 20% progressing to cirrhosis or hepatocellular carcinoma [4]. NASH remains the leading cause of liver disease among individuals awaiting liver transplants in the United States. The underpinning premise linking NAFLD to metabolic syndromes, incorporating insulin resistance, dyslipidemia, and type 2 diabetes mellitus, has garnered scholarly attention [3].

Conventionally, percutaneous liver biopsy serves as the gold standard for liver disease diagnosis [5]. It affords confirmation of steatosis and quantification of fibrosis, ballooning, and lobular inflammation. Noteworthy scoring systems, such as the SAF score and NAFLD activity score, evaluate disease severity post-biopsy by scrutinizing fibrosis, activity, and steatosis parameters, yielding objective and comprehensive insights [5]. However, inherent limitations encompass tissue sampling variability, inter-observer discrepancies, invasiveness-associated risks, and discomfort [6]. Additional limitations of such scoring systems include sampling error, resource-intensive nature, and invasiveness [6]. Serial biopsies, though informative, present several substantial challenges. These include their invasiveness, the associated risks, and the patient's reluctance to undergo multiple invasive procedures over time. Moreover, serial biopsies are resource-intensive and may not accurately represent the dynamic nature of the disease, potentially missing temporal changes. In turn, this limitation has a significant impact on patient care. The inability to perform serial biopsies hampers our ability to continuously monitor disease progression, assess the efficacy of treatments, and adapt patient care plans accordingly. It underscores the urgency of developing non-invasive methodologies for diagnosing, screening, and monitoring fatty liver disease [7]. These non-invasive approaches are essential not only for reducing the burden on patients but also for ensuring that healthcare providers have access to real-time, comprehensive data for effective disease management and personalized treatment strategies.

Artificial intelligence (AI), a field of computer science that focuses on the development of algorithms and models enabling machines to perform tasks that typically require human intelligence, has become a transformative force increasingly integrated into imaging and clinical screening systems to bolster diagnostic accuracy [3]. This is especially true for machine learning and deep learning, which are subsets of AI. The past decade has witnessed AI's prowess in discerning patterns and correlations within vast datasets across medical disciplines, thus making it aptly poised for advancing disease diagnostics [8].

The existing literature on AI applications in diagnosing fatty liver disease highlights several important aspects. In a meta-analysis to evaluate the diagnostic accuracy and reliability of ultrasonography for detecting fatty liver [9], ultrasonography was found to be a reliable method for diagnosing fatty liver, but there may be limitations in its accuracy. Barre et al. reviewed the application of AI in gastroenterology and hepatology and discussed its potential in aiding diagnosis and prognosis [10]. They emphasized the need for further randomized, controlled studies to validate AI techniques. Furthermore, Lin et al. compared the diagnostic criteria for NAFLD and metabolic-associated fatty liver disease (MAFLD) in the real world [11]. They highlighted the novelty of the MAFLD concept and the need for validation in real-world settings, which extends to diagnosis using AI.

Moreover, Wai et al. reviewed the confounding factors of non-invasive tests for NAFLD [12]. They emphasized the importance of considering these factors when interpreting test results. Finally, Decharatanachart et al. conducted a systematic review and meta-analysis on the application of AI in chronic liver diseases, reporting that AI techniques have the potential to diagnose liver fibrosis [13]. Overall, the existing literature highlights the potential of AI in diagnosing fatty liver disease, but further research is needed to validate its effectiveness and address the limitations of current diagnostic methods. Our study attempts to fill in this gap in the literature.

Notably, AI applications have played a pivotal role in liver disease management, encompassing the prognostication of liver decompensation, facilitating transplant recipient selection, and predicting transplant complications and survival [8]. The integration of AI extends across various domains in healthcare, including electronic health records, digital pathology, and medical imaging. Within the field of medical imaging, AI augments diagnostic precision, particularly in the context of fatty liver diseases such as NAFLD. AI-driven diagnostic systems have exhibited remarkable accuracy, aided by a spectrum of commonly employed algorithms, including support vector machines (SVMs), convolutional neural networks (CNNs), multilayer perceptron, fuzzy Sugeno, and probabilistic neural networks (PNNs), among others. Despite these advancements, the existing literature lacks comprehensive systematic reviews or meta-analyses that synthesize the efficacy of AI-assisted diagnostic systems, especially concerning fatty liver diseases using imaging data. In line with this perspective, our systematic review aims to elucidate the performance and viability of AI-assisted systems in diagnosing fatty liver disease via imaging data.

## Review

Methodology

Search Strategy

We followed the Preferred Reporting Items for Systematic Reviews and Meta-Analyses (PRISMA) framework for our study's methodology. Our goal was to find research studies that used AI to diagnose and categorize fatty liver disease using imaging data. To do this, we looked for relevant articles in major databases like Google Scholar and Embase. We focused on articles published between January 2010 and May 2023, avoiding older ones to stay up to date with current AI techniques.

We used a targeted search strategy with specific keywords and Boolean operators to ensure comprehensive results. Our search terms included "fatty liver disease" AND ("AI" OR "artificial intelligence") AND ("diagnos*" OR "categoriz*") AND ("imaging data" OR "hepatic imaging") AND ("cirrhosis" OR "fibrosis" OR "steatohepatitis" OR "NASH" OR "NAFLD" OR "MAFLD") AND ("deep learning" OR "machine learning" OR "neural network"). The protocol for this review was not registered. This decision was made to allow flexibility in responding to the rapid emergence of relevant research in the field and undergoing this research effort in a very quick manner, ensuring our systematic review remains up-to-date and comprehensive. Table 1 shows the full search strategy utilized in the search.

**Table 1:** Comprehensive search strategy for identifying studies on AI-assisted diagnosis and categorization of fatty liver disease using imaging data

Database	Search terms
Google Scholar	("fatty liver disease" AND ("AI" OR "artificial intelligence") AND ("diagnos*" OR "categoriz*") AND ("imaging data" OR "hepatic imaging") AND ("cirrhosis" OR "fibrosis" OR "steatohepatitis" OR "NASH" OR "NAFLD" OR "MAFLD") AND ("deep learning" OR "machine learning" OR "neural network")) AND ("2010-01-01" [Date - Publication]: "2023-05-31" [Date - Publication])
Embase	("fatty liver disease"/exp OR "fatty liver disease" OR "hepatic steatosis" OR "non-alcoholic fatty liver disease" OR "NAFLD" OR "NASH" OR "non-alcoholic steatohepatitis" OR "MAFLD" OR "AI" OR "artificial intelligence") AND ("diagnos*"/exp OR "categoriz*"/exp OR "diagnos*" OR "categoriz*") AND ("imaging data"/exp OR "imaging data" OR "hepatic imaging") AND ("cirrhosis" OR "fibrosis" OR "steatohepatitis" OR "deep learning" OR "machine learning" OR "neural network") AND ("2010-01-01" : "2023-05-31")

Inclusion and Exclusion Criteria

To provide a clear and coherent focus within our study, we implemented specific inclusion and exclusion criteria. In selecting studies for inclusion, we prioritized those employing AI techniques in the context of fatty liver disease diagnosis. These selected studies were required to present sufficient data, specify the AI classification used, and provide comprehensive details about their diagnostic methodology. This emphasis on clear descriptions aimed to ensure the transparency and reliability of the studies included in our review.

Conversely, our exclusion criteria were designed to maintain the rigor and quality of the review. We excluded studies that did not report the desired outcomes or those with insufficient data. Additionally, studies lacking vivid descriptions of validation cohorts and the characteristics of training or validation methods were omitted. The decision to exclude non-English studies was made to ensure the accessibility of our review to a wider audience and to minimize potential language-related interpretation issues.

We also excluded abstracts, conference proceedings, editorials, and reviews that lacked sufficient data regarding source image features or the study population. Furthermore, studies without the requisite data for constructing the 2x2 table were excluded to maintain the analytical robustness of our review. These criteria were carefully applied to ensure the integrity and quality of our study, with the goal of providing a comprehensive and reliable analysis of AI-based diagnostic methods for fatty liver disease. For a comprehensive outline of the detailed inclusion and exclusion criteria, please consult Table 2.

**Table 2:** Detailed inclusion and exclusion criteria for the selection of studies on AI-assisted diagnosis and categorization of fatty liver disease using imaging data

Inclusion criteria	Exclusion criteria
Studies employing AI for fatty liver disease diagnosis	Studies not reporting desired outcomes or having insufficient data
Studies providing sufficient data, specifying AI class, and detailing diagnostic method	Studies lacking clear descriptions of validation cohorts and training/validation methods
Studies utilizing either definition for fatty liver disease (biopsy-based definition or imaging characteristics)	Studies reported in languages other than English
	Abstracts, conference proceedings, editorials, and reviews lacking adequate data on source image features or study population
	Studies without adequate data for constructing the 2x2 table

Data Extraction

The screening of titles and abstracts was conducted by the main author to identify studies for further assessment in the full-text review phase. Following the completion of the screening process, the subsequent step involved data extraction, which was undertaken independently by the author and cross-verified as necessary. In cases where uncertainty arose regarding the eligibility of specific data for inclusion in the study, a comprehensive cross-checking procedure was revisited to ensure a thorough examination. The extracted data encompassed various specific details, including validation cohorts, training and validation method particulars, study design (whether retrospective or prospective cohort), study location, publication year, and authorship. Additionally, the data extraction process captured specificity and sensitivity values. In instances where investigations encompassed multiple AI classifiers, the focus was directed toward the AI classifier demonstrating optimal performance, as assessed by the highest area under the curve or superior accuracy.

Results

Literature Search

**Figure 1:**
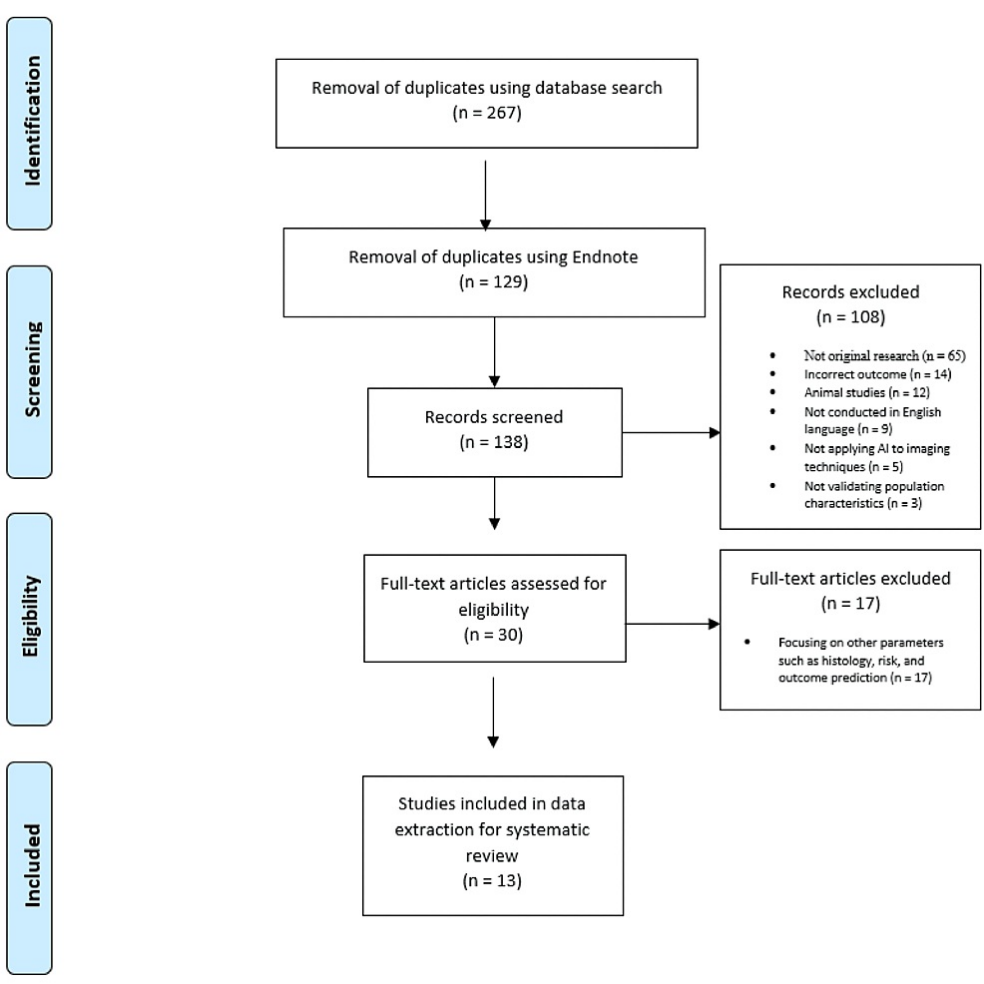
PRISMA study selection flow diagram

Retrieved articles were entered into a reference software manager, and duplicates (n = 129) were removed. As illustrated in Figure 1, the titles and abstracts of all search results (n = 267) were independently screened by the investigators for relevance. Following duplication removal, of the remaining 138 articles, some were not original research (such as editorials and reviews) (n = 65), with other studies examining diseases other than any of the types of fatty liver disease (n = 14), studies that had focused on animal research (n = 12), and those that had not been documented in the English language (n =9). Some studies also failed to report validation population characteristics or the desired outcomes (n = 3), while others did not apply AI to imaging data to diagnose fatty liver disease (n = 5). Some studies focused on other parameters like histology, risk, and outcome prediction (n = 17). Eventually, 13 articles were found that employed AI-assisted ultrasonography. Ultrasound was predominantly employed in almost all the selected studies as the primary imaging modality. This preference for ultrasound was primarily due to its affordability and accessibility. Given its cost-effectiveness and widespread availability, ultrasound served as the imaging method of choice in the majority of these studies. Additionally, several of the selected studies harnessed the power of artificial neural networks (ANNs) to enhance the diagnostic capabilities of ultrasound. Some of the types of AI that the selected studies embraced included ANNs, CNNs, SVMs, and multiple AI models. ANNs and CNNs are specialized neural networks designed for image analysis, while SVM is a machine learning algorithm used for classification and regression tasks. The PRISMA protocol depicting this literature search process is highlighted in summary form, as shown in Figure 1.

Table 3 offers a summary of the findings regarding the characteristics of the studies and their associated findings.

**Table 3:** Summary of results from included studies on AI-assisted diagnosis and categorization of fatty liver disease using imaging data CNN: convolutional neural network, PNN: probabilistic neural network, SVM: support vector machine, DT: decision tree

Author	Year of publication	Country/research setting	Sample size	AI classifier	Diagnostic method	Sensitivity as extracted from the article	Specificity as extracted from the article	Diagnostic accuracy as extracted from the article
Rhyou and Yoo [14]	2021	Korea	3200	CNN (Inception v3)	Ultrasound	99.78%	100%	99.91%
Acharya et al. [15]	2016	Singapore	100	Fuzzy Sugeno	Ultrasound	100%	100%	100%
Gummadi et al. [16]	2020	U.S	905	CNN	Ultrasound	88.6%	95.3%	92.3%
Acharya et al. [17]	2016	Malaysia	150	PNN	Ultrasound	96%	100%	97.33%
Byra et al. [18]	2018	Poland	550	CNN	Ultrasound	100%	88.2%	96.3%
Acharya et al. [19]	2012	Singapore	100	DT	Ultrasound	88.9%	100%	93.3%
Zamanian et al. [20]	2021	Iran	550	SVM	Ultrasound	97.2%	100%	98.64%
Neogi et al. [21]	2018	India	340	PNN	Ultrasound	100%	87%	99%
Zhang et al. [22]	2019	China	500	CNN	Ultrasound	83%	95%	90%
Han et al. [23]	2020	U.S	204	CNN	Ultrasound	97%	94%	96%
Reddy et al. [24]	2018	India	157	CNN	Ultrasound	95%	85%	90.6%
Ma et al. [25]	2018	China	10508	Bayesian network model	Ultrasound	67.5%	87.8%	83%
Gaber et al. [26]	2022	Egypt	300	J48 algorithm, voting-based classifier	Ultrasound	97.05%	94.44%	95.71% (voting-based classifier), 93.12% (J48 algorithm)

The studies included in this comprehensive review collectively underscore the transformative potential of AI integration in enhancing the accuracy and reliability of ultrasound analysis for fatty liver disease diagnosis. These selected studies reveal that the incorporation of AI into ultrasound analysis not only improves overall performance but also serves as an effective means to mitigate potential human errors, thereby enhancing diagnostic precision. Notably, the remarkable capacity of AI-assisted ultrasound for the early detection of steatosis stands out as a critical advancement in the field, offering the potential for timely intervention and improved patient outcomes.

In our comprehensive review of various studies, we observed a wide array of AI classifiers being employed, predominantly focused on ultrasound diagnostics. The classifiers ranged from CNN, with specific instances like Inception v3, to more complex models such as fuzzy Sugeno CNN, PNN, decision tree (DT), SVM, Bayesian network models, the J48 algorithm, and voting-based classifiers. This diversity reflects the dynamic and evolving nature of AI applications in medical imaging.

Delving into the methodologies of specific studies, Rhyou et al. employed a meticulous approach where medical experts annotated ultrasound images, categorizing them into four levels of steatosis: normal, mild, moderate, or severe. Subsequently, the dataset was divided into a 6:2:2 ratio for training, validation, and testing, ensuring a balanced approach for model training and performance evaluation. This methodological rigor aids in mitigating the risk of overfitting, a critical aspect of machine learning models [14].

Contrasting this, Zamanian et al. adopted a more comprehensive approach for network training by utilizing multiple image collections rather than the single image classification method prevalent in earlier studies. This broader approach likely enhances the model's ability to generalize better across various cases, an important consideration in the practical application of AI in medical diagnostics [20].

Furthermore, it is crucial to acknowledge the dependency of AI systems on their training and data quality. The effectiveness of these systems in diagnosing conditions like steatosis is heavily influenced by the data they are trained on, which was a key factor across all studies. Moreover, the role of the examiner is paramount in ensuring the accuracy of AI classifiers. The skill with which examiners provide relevant and quality material for AI training cannot be overstated and is a consistent theme across the reviewed literature, highlighting the synergy between human expertise and AI in advancing medical diagnostics.

Sample sizes in the studies ranged from 100 in a Singapore-based study to an impressive 10,508 in a China-based study, reflecting a diverse spectrum of research populations and allowing for a comprehensive assessment of AI's effectiveness across different sample sizes. Furthermore, the AI classifiers employed in these studies exhibited remarkable diversity, including DTs, PNNs, and CNNs, underscoring the versatility of AI techniques in fatty liver disease diagnosis. These studies were conducted over a span of several years, from 2016 to 2022, demonstrating the continued evolution of AI methodologies in this field.

In addition to the diversity in sample sizes and AI classifiers, the studies in this review were conducted across diverse populations and geographical regions, encompassing Korea, the United States, Malaysia, Poland, Singapore, Iran, and India. This diversity in study locations and patient demographics enriches the generalizability of the findings and highlights the global relevance of AI-assisted fatty liver disease diagnosis.

Despite these promising advancements, there remain several avenues for further refinement that warrant scholarly exploration. To address the prevailing challenges faced by current AI classifiers, a concerted effort is needed to ameliorate specific aspects of the analysis process.

Firstly, enhancing image accuracy upon extraction from expansive datasets is crucial. This challenge involves ensuring the quality and fidelity of input data to maintain the integrity of AI-assisted diagnostics. Secondly, the reduction of dimensionality for improved computational efficiency is paramount. Simplifying complex datasets and algorithms can significantly impact the speed and scalability of AI models, making them more practical and accessible for clinical use. Thirdly, mitigating issues related to computational time is essential. Improving the efficiency and speed of AI algorithms is a key consideration for real-time or near-real-time diagnosis. Lastly, bridging the semantic gap and addressing speckle noise in ultrasound images are critical steps toward harnessing the full potential of AI-based analysis, ultimately contributing to more reliable and precise diagnoses.

The semantic gap refers to the disparity between the raw data in medical images and the clinical understanding required for an accurate diagnosis. This gap can hinder the performance of AI-based systems as they struggle to comprehend the nuanced features of pathology. For example, when interpreting ultrasound images, AI may encounter difficulties in distinguishing between benign and malignant lesions due to the subtleties in tissue appearance. Researchers are actively working on semantic segmentation models and natural language processing techniques to bridge this gap, enabling AI to provide more clinically relevant insights. Speckle noise, an inherent interference pattern in ultrasound images, can introduce artifacts and affect the accuracy of AI analysis. For instance, it may lead to false positives or negatives in the diagnosis of fatty liver disease. Solutions to mitigate speckle noise include advanced image processing techniques, such as despeckling filters and deep learning-based noise reduction algorithms. These strategies are pivotal in enhancing image quality and, subsequently, the reliability of AI-assisted diagnosis. Addressing these challenges in bridging the semantic gap and mitigating speckle noise is paramount to realizing the full potential of AI-based analysis. By developing and implementing effective solutions, researchers can contribute to more reliable and precise diagnoses, ultimately improving patient care and clinical decision-making.

Furthermore, the exploration of parameter effects and optimization strategies is vital to driving continuous improvements in model performance. As the field advances toward enhanced accuracy and clinical applicability, the acquisition of more extensive and diverse datasets, encompassing inputs from various operators and patients across different healthcare centers, becomes pivotal. Additionally, expanding the consideration of disease stages and employing advanced image improvement techniques prior to analysis could yield enriched insights, further enhancing the diagnostic capabilities of AI in the context of fatty liver disease.

In summary, the findings presented in this review not only highlight the significant achievements in AI-assisted fatty liver disease diagnosis but also underscore the potential areas for refinement within this rapidly evolving field. The integration of AI holds great promise for revolutionizing clinical practice by improving diagnostic accuracy, reducing errors, and facilitating early disease detection. Continued research efforts aimed at addressing existing challenges and optimizing AI-based diagnostic models will play a crucial role in maximizing the impact of AI in the diagnosis and management of fatty liver disease.

Discussion

This systematic review delved into diverse AI-assisted techniques for the diagnosis of fatty liver disease. The results unveiled a compelling narrative, showcasing the remarkable performance of AI-enhanced ultrasonography in diagnosing various types of fatty liver disease, particularly NAFLD. Notably, the majority of the studies reviewed reported very high diagnostic accuracy, with figures ranging from 83% to 100%. This range not only underscores the efficiency of AI-enhanced ultrasonography but also highlights its reliability. Moreover, these studies consistently demonstrated high specificity and sensitivity, reinforcing the potential of AI as a powerful tool in the early and accurate diagnosis of fatty liver diseases. Furthermore, this diagnostic prowess was complemented by the identification of relatively low heterogeneity across a substantial portion of the studies.

However, it's important to recognize that variations in clinical input did contribute to instances of elevated heterogeneity. These variations may stem from differences in patient populations, clinical settings, or diagnostic methodologies across the included studies. Such heterogeneity is significant for the interpretation of study results, as it highlights the need for a nuanced understanding of AI's performance in diverse clinical scenarios. These variations underscore the importance of considering the context in which AI-assisted diagnostics are applied and the potential impact of these factors on diagnostic outcomes. Amidst these nuanced findings, a consistent theme emerged: when AI is seamlessly integrated into ultrasonography, the diagnostic landscape of fatty liver disease experiences a significant uplift. A crucial aspect to bear in mind is the widespread availability of ultrasonography across healthcare facilities and hospitals, characterized by its non-invasiveness and cost-effectiveness [3]. However, the dependence on user interpretation in ultrasonography analysis could potentially introduce intra- and inter-observer variations, ultimately challenging the reliability of conventional ultrasonography in diagnosing fatty liver disease, including NAFLD. This systematic review substantiates the notion that the fusion of AI with ultrasonography effectively minimizes human-related errors, culminating in a promising enhancement of diagnostic performance. Consequently, these findings substantiate the robustness and advantages inherent in the utilization of AI-assisted ultrasonography. Nevertheless, the ongoing pursuit of clinical validation necessitates randomized controlled trials pitting conventional imaging modalities against AI-assisted systems to rigorously validate performance disparities.

NAFLD diagnosis, particularly in the identification of fibrosis and NASH, holds significant clinical importance. Identifying the degree of fibrosis and the presence of NASH is pivotal for tailoring patient management strategies and determining the appropriate interventions. NASH, characterized by inflammation and hepatocellular injury, signifies a higher risk of disease progression and complications, making its accurate diagnosis crucial for timely therapeutic measures. AI's diagnostic acumen in identifying NASH appears encouraging, accompanied by an acceptable level of sensitivity. However, it's pertinent to acknowledge that the observed heterogeneity, albeit influenced in part by varying populations and diagnostic methods, prompts reflection on the scarcity of studies available. The limited study pool underscores the imperative for increased research efforts, fostering an expanded repertoire of investigations to enable more comprehensive and reliable analyses.

In the pursuit of advancing fatty liver disease diagnostics, AI stands as a catalyst, offering insights into the potential of combining cutting-edge technology with clinical practice. The avenues for AI's integration are abundant, promising substantial improvements in accuracy and clinical decision-making across critical areas such as early disease detection, treatment selection, and patient management. The outcomes of this systematic review lay a foundation, both affirming current advancements and underscoring the critical necessity for continued research and robust validation efforts in the dynamic landscape of AI-enhanced diagnostics.

Strengths and Limitations

This systematic review leverages a rigorous methodology based on the PRISMA framework, ensuring comprehensive coverage of the relevant literature on AI-assisted diagnosis of fatty liver disease. The inclusion of major databases, such as Google Scholar and Embase, enhances the breadth of the literature search. The detailed search strategy, as presented in Table 1, facilitated the identification of a diverse range of studies addressing the intersection of AI and fatty liver disease diagnosis. Notably, this diversity includes studies from various geographical regions, adding an additional layer of depth and context to our analysis as well as improving the generalizability of the findings. The systematic approach employed for data extraction and analysis reinforces the reliability and validity of the findings.

The review's focus on AI integration with ultrasonography contributes to its clinical relevance, given the widespread use of ultrasonography in healthcare settings. By highlighting the potential of AI in enhancing the accuracy and reliability of ultrasonography-based fatty liver disease diagnosis, this review underscores the imminent translational impact of AI technologies.

Despite the comprehensive methodology employed, this review is not immune to certain limitations. Firstly, the limited number of studies retrieved from the literature review is a limitation to the level of evidence synthesized. The identified studies predominantly concentrated on AI applications in ultrasonography, potentially limiting the generalizability of the findings to other imaging modalities. The lack of studies directly comparing AI-assisted systems with conventional imaging modalities restricts the ability to draw conclusive performance comparisons. Furthermore, heterogeneity observed across studies, attributed to varying clinical inputs and methodologies, introduces a degree of complexity in the interpretation of the collective findings.

Additionally, it's crucial to acknowledge that the exceptionally high accuracy rates reported in some studies may raise concerns about overfitting, wherein AI models perform exceedingly well on the training data but may not generalize optimally to new, unseen data. This aspect warrants careful consideration when interpreting the results. Moreover, the relatively limited number of studies addressing specific aspects, such as the identification of NASH, warrants cautious interpretation and calls for additional research efforts in these domains.

The review's scope extends only to studies published in English, which may introduce potential language bias and exclude relevant non-English publications. Finally, while efforts were made to encompass a range of studies, it is plausible that some studies within the rapidly evolving field of AI-assisted diagnosis were not captured in the search.

Future Research

Moving forward, this systematic review spotlights crucial avenues for future research in the domain of AI-assisted diagnosis of fatty liver disease. While the integration of AI with ultrasonography has demonstrated remarkable potential, the need for head-to-head comparisons with conventional imaging modalities through randomized controlled trials remains paramount. Rigorous validation efforts are essential to not only corroborate the superiority of AI-assisted systems but also to ascertain the specific contexts in which these systems excel. However, it's essential to acknowledge the potential challenges and ethical considerations inherent in such trials, including issues related to patient consent, data security, and ensuring equitable access to AI-assisted diagnostic technologies.

Moreover, as AI's diagnostic prowess expands beyond NAFLD diagnosis, the intricate landscape of fibrosis and NASH assessment warrants further exploration. The heterogeneous findings observed in this review underscore the necessity for a larger and more diverse pool of studies to provide a robust foundation for comprehensive analyses. As the field advances, it is imperative to consider not only diagnostic accuracy but also the integration of AI into clinical workflows and its potential impact on patient outcomes.

Additionally, the refinement of AI algorithms to address the challenges of image accuracy, dimensionality reduction, computational efficiency, and noise reduction remains a crucial research frontier. Future studies should explore the optimization of AI models through the examination of parameter effects, leveraging expansive datasets from diverse patient populations and healthcare centers.

## Conclusions

In synthesis, this systematic review underscores the promise of AI-assisted diagnosis for various types of fatty liver disease, including NAFLD, through the integration of imaging data and diverse diagnostic methods. Across a diverse range of studies with varying sample sizes, AI classifiers, and geographical locations, AI consistently demonstrated impressive diagnostic accuracy, specificity, and sensitivity. Notably, neural network-based AI systems exhibited superior performance, emphasizing the role of advanced machine learning techniques in achieving remarkable diagnostic precision.

The encouraging outcomes highlight AI's potential in identifying NASH, fibrosis, and steatosis, though the limited number of studies in certain areas necessitates cautious interpretation. Future large-scale studies are vital to substantiate the positive impact of AI-assisted diagnosis in fatty liver disease, paving the way for its transformative integration into clinical practice. Challenges and considerations in conducting these large-scale studies include the need for standardized AI algorithms, diverse patient populations, and real-world clinical settings for validation. Overcoming these challenges will be essential in ensuring the seamless integration of AI into the broader landscape of clinical practice and healthcare delivery, ultimately enhancing the precision and accessibility of fatty liver disease diagnosis.
